# Identification of a novel somatic mutation of *POU6F2* by whole‐genome sequencing in prolactinoma

**DOI:** 10.1002/mgg3.1022

**Published:** 2019-11-06

**Authors:** Yazhou Miao, Chuzhong Li, Jing Guo, Hongyun Wang, Lei Gong, Weiyan Xie, Yazhuo Zhang

**Affiliations:** ^1^ Department of Cell Biology Beijing Neurosurgical Institute Capital Medical University Beijing China; ^2^ Department of Neurosurgery Beijing Tiantan Hospital Affiliated to Capital Medical University Beijing China

**Keywords:** mutation, *POU6F2*, prolactinoma, whole‐genome sequencing

## Abstract

**Background:**

Pituitary adenomas (PAs) are one of the most common intracranial tumors; approximately half of PAs are prolactin (PRL)‐secreting PAs (prolactinomas). The genetic alterations prevalent in prolactinomas are unknown.

**Methods:**

Here, we present a patient with an extremely aggressive and giant prolactinoma accompanied by serious destruction of the surrounding bone mass. This patient exhibited resistance to dopaminergic drugs. Through whole‐genome sequencing, we identified two novel somatic mutations in the *POU6F2* gene (NM_001166018.2: c. 839 C>T; NM_001166018.2: c. 875A>G).

**Results:**

This report is the first to identify these somatic mutations in the *POU6F2* gene in a prolactinoma. We found that these two mutations obviously decreased the expression level of POU6F2. Inhibition of POU6F2 activity increased the cell proliferation and PRL secretion in rat pituitary cells, but proliferation and PRL secretion were decreased in cells with POU6F2 overexpression.

**Conclusions:**

*POU6F2* might play a crucial role in the development of prolactinomas and may be a promising target for developing new therapies against prolactinomas.

## Graphical abstract

It is the first report of somatic mutation in POU6F2 gene in a prolactinoma. We found that these two mutations obviously decreased the expression level of POU6F2. Inhibition of POU6F2 activity increased the cell proliferation and prolactin (PRL) secretion in rat pituitary cells, the cell proliferation and secretion of PRL were decreased when POU6F2 overexpression was achieved.

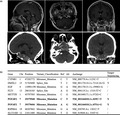

## 1 INTRODUCTION

Prolactinomas account for 32%–66% of pituitary adenomas (PAs) and present as amenorrhea, loss of libido, galactorrhoea, and infertility in women and as loss of libido, erectile dysfunction, and infertility in men; they are generally treated with the dopamine agonists cabergoline and bromocriptine (Molitch, 2017). Although prolactinomas are considered benign tumors, due to the drug resistance of some patients and the unique hormonal disorders caused by prolactinomas, exploration of the key genes in prolactinoma tumorigenesis is necessary.

Several genetic alterations have been described in PAs. For example, guanine nucleotide‐binding protein α subunit (*GNAS*) alterations are found in somatotroph adenomas (Landis et al., 1989) and ubiquitin‐specific protease 8 (*USP8*) alterations are found in corticotroph adenomas (Ma et al., 2015). Germline AIP mutations are found in sporadic pituitary adenoma patients with a family history of this disease (Cazabat et al., 2012; Chahal, 2011). Germline mutations in *CDH23*, which encodes Cadherin‐Related 23, are associated with both familial and sporadic PAs (Zhang et al., 2017). Some PAs have mutations in the menin gene, which can cause multiple endocrine neoplasia type 1. These PAs tend to be mainly prolactin adenomas and show stronger aggressiveness (Caimari & Korbonits, 2016).

Although most pituitary tumors are benign, some PAs have a tendency toward malignant growth, and even attack the surrounding bone. Thus, we performed whole‐genome sequencing on matched tumor and normal blood samples from a patient with aggressive prolactinoma with severe invasion and surrounding bone destruction, aiming to identify somatic genetic alterations. We found that prolactinomas have a low mutational burden, as reflected by their benign nature (Caimari & Korbonits, 2016; Song et al., 2016). In addition, we identified novel somatic mutations in the POU domain, class 6, transcription factor 2 (*POU6F2*) gene (OMIM, *609062, NM_001166018.2: c. 839 C>T; c. 875A>G). POU family members are transcriptional regulators and *POU6F2* is a tumor suppressor involved in the predisposition to Wilms’ tumor (Perotti et al., 2004). The MMQ cell line, a rat prolactinoma cell line (Judd et al., 1988), was used to explore the role of *POU6F2* in prolactinomas. We used plasmids and small interfering RNA (siRNA) to overexpress and knock down POU6F2, and found an increase in viability and prolactin (PRL) secretion were decreased in MMQ cells with POU6F2 overexpression. In contrast, in MMQ cells with *POU6F2* knockdown, viability and PRL secretion were increased. Our study suggests that *POU6F2* is also a tumor suppressor in prolactinomas and is a potential molecular therapeutic target for the control of prolactinomas.

## 2 MATERIALS AND METHODS

### 2.1 Editorial policies and ethical considerations

All procedures performed on samples were approved by the Ethics Committee of Beijing Tiantan Hospital. The patient signed an informed consent.

### 2.2 Patient

The patient in this study was a 43‐year‐old male in whom preoperative magnetic resonance imaging (MRI) showed a tumor volume of 46.6 × 62.3 × 21.4 mm^3^ and a Knosp grade of IV. The maximum PRL level before surgery was 5,453 ng/ml, and was reduced to 1068 ng/ml after three months of oral bromocriptine treatment at a dosage of 15 mg/day, with no significant tumor shrinkage. The patient had undeveloped secondary sexual characteristics, loss of libido, erectile dysfunction, galactorrhoea, and infertility, and he underwent neuroendoscopic pituitary adenoma resection in Tiantan Hospital. The postoperative PRL level was reduced to 273 ng/ml, and postoperative pathological staining showed positive PRL, but negative results for the other hormones. Tissue samples and peripheral blood samples were obtained and stored at Beijing Neurosurgical Institute, Beijing, China. All of the main clinical information is summarized in Table S1.

### 2.3 Whole‐genome sequencing and Sanger sequencing validation

Whole‐genome sequencing was performed on DNA from tumor and matched blood samples. The mean tumor purity was estimated to be greater than 90%. A sequencing library was constructed using a Truseq Nano DNA HT Sample Prep Kit (FC‐121‐4003, Illumina) and sequenced on the Illumina HiSeq X platform to an average depth of 50× for tumor samples and 30× for matched blood samples, with 99% coverage of the known genome. DNA sequencing and integrative analysis of the data in this study were completed by Novogene Bioinformatics Institute. To identify the biallelic mutation, the PCR product was gel purified and cloned into the pGEM^®^ T vector (Promega). Plasmids were isolated from single colonies for the identification of *POU6F2* mutations and DNA sequencing.

### 2.4 Cell culture and cell transfection

The MMQ cell line was purchased from the American Type Culture Collection (ATCC) cell bank. Cells were cultured in ATCC‐formulated F‐12K medium (Invitrogen) containing 2.5% foetal bovine serum (Gibco) and 15% horse serum (Gibco) in a 37°C incubator with a humidified atmosphere of 95% air and 5% CO_2_. HEK 293 cells were cultured in the same incubator in Dulbecco's modified Eagle medium supplemented with 10% FBS. Cultures were fed every other day. MMQ cells were transfected with siRNA and plasmid vector using Lipofectamine^®^ 3000 (Thermo Fisher Scientific). The pCMV6‐AC‐GFP–*POU6F2* (RG228521) construct was purchased from OriGene Technologies. Mutant *POU6F2* (*POU6F2* 280/292A) was generated with a QuickChange site‐directed mutagenesis kit (Stratagene). The sequences of *POU6F2* siRNA are shown in Table S2.

### 2.5 Immunofluorescence

Cells in culture dishes were washed with PBS three times, fixed with 4% paraformaldehyde for 10 min, and washed with PBS three times for 5 min each. The stained section was examined with a Leica TCS SP5 II confocal microscope.

### 2.6 Protein extraction and western blot analysis

Collected cells were washed with 1× PBS buffer, prepared with RIPA buffer supplemented with protease/phosphatase inhibitor cocktail, and centrifuged at 12,000 r/min for 5 min at 4°C to yield the total protein extract in the supernatants. The protein concentration was measured with a BCA assay kit (Beyotime Institute of Biotechnology) according to the manufacturer's protocol. Equal amounts of protein were separated by 8% SDS‐PAGE and subsequently transferred to polyvinylidene difluoride membranes (Millipore). Membranes were blocked with 5% nonfat milk in Tris‐buffered saline with Tween^®^20 (TBST) for approximately 1 hr, followed by incubation with primary anti‐GAPDH (1:5,000; G8795, Sigma‐Aldrich), anti‐β‐actin (1:5,000; A1978, Sigma‐Aldrich), and rabbit polyclonal anti‐POU6F2 (1:1,000, TA351549, OriGene Technologies) antibodies overnight at 4°C. After washing with TBST, membranes were incubated with horseradish peroxidase conjugated secondary antibody (Cell Signaling Technology) at room temperature for 1 hr. ImageJ (NIH) was used to quantify the protein band densities.

### 2.7 MTS assay

Cells were seeded into 96‐well plates at a density of 1 × 10^4^ cells/well in 100 μl of cell culture medium for 12 hours and were then transiently transfected with the indicated plasmids and short interfering RNA. Ninety‐six hours after transfection, 20 μl 3‐(4,5‐dimethylthiazol‐2‐yl)‐5‐(3‐carboxymethoxyphenyl)‐2‐(4‐sulfophenyl)‐2H‐tetrazolium (MTS, G3580, Promega) buffer was added to each well, and the cells were then incubated at 37°C for another 3 hours. The absorbance of each well was measured at 490 nm. For each experimental replicate, we set up five technical replicates per set.

### 2.8 ELISA

PRL protein levels were determined using a rat PRL ELISA kit from BioVision (K4688‐100) according to the manufacturer's instructions. Briefly, MMQ cell lines were harvested 72 hr after treatment with siRNA or plasmid. The total protein content of the cells was determined for standardization of PRL production with a BCA protein assay kit (Pierce Biotechnology). The culture supernatants were collected and normalized to the cell numbers. All experiments were performed more than three times.

### 2.9 Data availability statement

All sequence data were submitted to the Sequence Read Archive database (PRJNA509733).

### 2.10 Statistical analysis

Experimental data are reported as the mean ± SEM (standard errors of the mean) of at least three independent experiments, as indicated in the corresponding figure legends and methods. Data were analyzed with one‐way ANOVA followed by the Kruskal–Wallis test.

## 3 RESULTS

### 3.1 Somatic *POU6F2* mutation in prolactinoma

**Figure 1:**
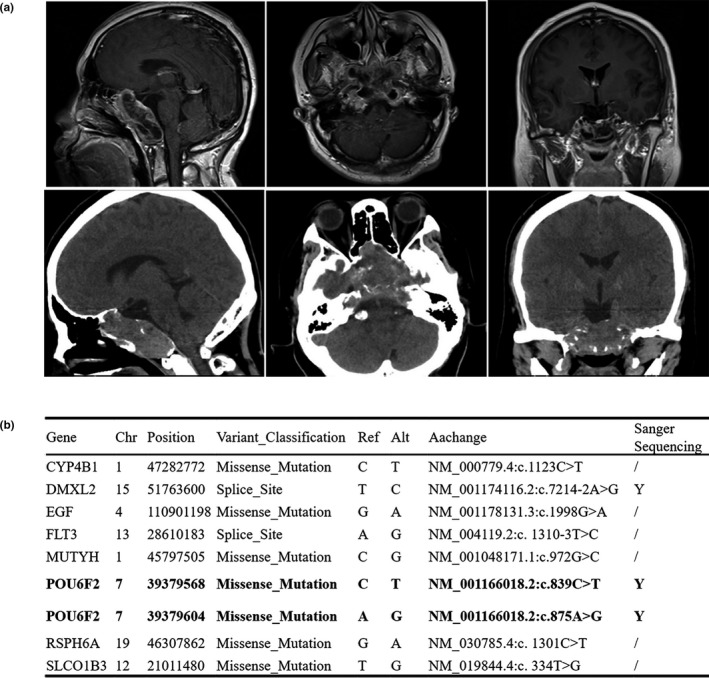
Preoperative images and mutation landscape of the prolactinoma. (a) Preoperative MRI and Computed Tomography (CT) demonstrate a giant adenoma with the invasion of the sphenoid sinus, clivus, and anterior skull base. (b) The mutation landscape of this prolactinoma. MRI, magnetic resonance imaging

**Figure 2:**
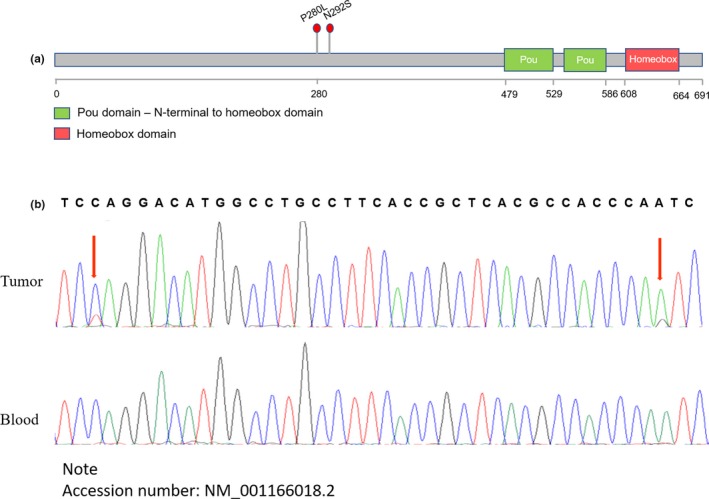
The mutation of *POU6F2*. (a) The Structure diagram of *POU6F2*. (b) *POU6F2* validation by Sanger sequencing. Arrows indicate mutated bases

The brain MRI showed a giant adenoma with invasion of the sphenoid sinus, clivus, and anterior skull base (Figure 1a). Whole‐genome sequencing of DNA extracted from the patient's tumor tissue and matched peripheral blood samples was performed to investigate somatic alterations. This assay revealed 9 somatic mutations (Figure 1b). No mutations were detected in any previously reported genes associated with PAs, including *GNAS*, *USP8* or *PRKACA*. However, we found two very close mutations in the *POU6F2* gene, one at position 280 and one at position 292, at the same time, they are biallelic mutations (Figure 2a, b). These results suggest that this gene may play a role in this prolactinoma patient.

### 3.2 Knockdown of *POU6F2* promotes cell viability and PRL secretion in the MMQ cell line

**Figure 3:**
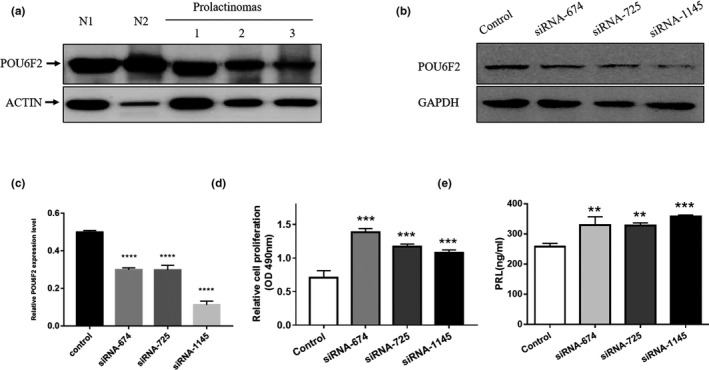
POU6F2 inhibition affect the cell proliferation and PRL secretion of MMQ cells. (a) Representative Western blots of POU6F2 in normal human pituitary and prolactinomas. Blots were reprobed with anti‐actin antibody to ensure equal loading. (b) Western blots of Pou6f2 in the MMQ cells which were transfected with siRNAs (negative control, Pou6f2‐rat‐674, Pou6f2‐rat‐725, Pou6f2‐rat‐1145). The expression of Pou6f2 in the siRNA groups was reduced compared with the control group. (c) The relative Pou6f2 expression level normalized by GAPDH. D, MTS experiments were used to determine whether Pou6f2 knockdown had an effect on MMQ cell viability. Compared with the control group, the siRNA groups have a significantly higher cell viability. (e) The PRL level in these MMQ cell groups, which were transfected with siRNAs (negative control, Pou6f2‐rat‐674, Pou6f2‐rat‐725, Pou6f2‐rat‐1145). Compared with the control group, the siRNA groups have a significantly higher level of PRL. **p < .01, ***p < .001, ****p < .0001, as compared with the control group (one‐way ANOVA followed by the Kruskal–Wallis test)

First, we investigated and confirmed POU6F2 protein expression in normal human pituitary glands and prolactinomas. Anti‐POU6F2 antibodies produced single bands in western blot analysis (Figure 3a). The β‐Actin was used as the internal control (Figure 3a).

To determine the role of POU6F2 expression in prolactinomas, we used siRNA targeting *POU6F2* mRNA in MMQ cells to inhibit the expression of POU6F2. The control siRNA and three targeting siRNAs (siRNA‐674, siRNA‐725, and siRNA‐1145) were separately transfected into MMQ cells with Lipofectamine 3000. After 72 hr, cellular proteins were extracted, and western blotting was conducted to verify whether the three siRNAs could knock down *POU6F2* (Figure 3b). According to the western blot image, the expression of POU6F2 in the siRNA groups was decreased compared with the control group. We then performed MTS assays to evaluate cell proliferation and found that cell viability was significantly higher in the siRNA groups than in the control group, suggesting that POU6F2 downregulation promotes MMQ cell viability (Figure 3c). To determine whether POU6F2 downregulation affects the ability of MMQ cells to secrete PRL, a PRL ELISA kit was used to measure the level of PRL in the supernatant of MMQ cells. The ELISA results showed that the level of PRL was significantly higher in the siRNA groups than in the control group and that POU6F2 downregulation promoted PRL secretion by MMQ cells (Figure 3d).

### 3.3 Overexpression of POU6F2 inhibits cell viability and PRL secretion in the MMQ cell line

**Figure 4:**
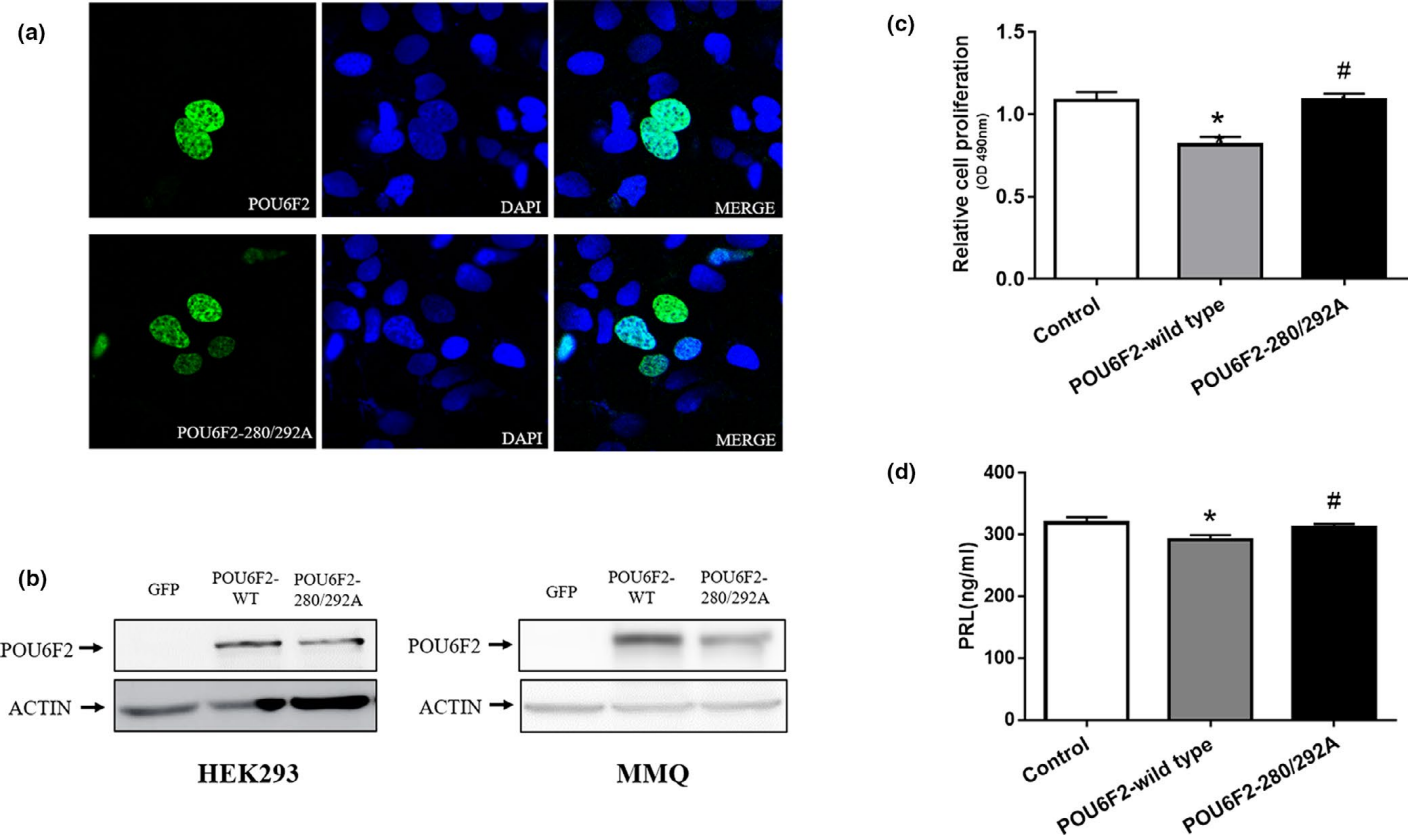
POU6F2 overexpression affects the cell proliferation and PRL secretion of MMQ cells. (a) Confocal images of HEK293 cells transfected with POU6F2 and POU6F2‐280/292A respectively. (b) Western blots of POU6F2 in the MMQ and HEK293 cells which are transfected with vector, POU6F2 wild‐type, and POU6F2‐280/292A, respectively. (c) MTS assay was used to determine the effect of POU6F2 on MMQ cell viability. Compared with the Pou6f2 group, the POU6F2‐280/292A group and the control group have a significantly higher cell viability. (d) The PRL level in these MMQ cell groups, which are transfected with vector, POU6F2 wild‐type, and POU6F2‐280/292A, respectively. Compared with the wild‐type group, the POU6F2‐280/292A group and the control group have a significantly higher level of PRL. *p < .05, as compared with the control group; ^#^ *p* < .05, as compared with POU6F2 group (one‐way ANOVA followed by Kruskal–Wallis test)

To confirm whether the *POU6F2* 280/292 mutations affect pituitary tumorigenesis, we constructed plasmids expressing wild‐type *POU6F2* and the *POU6F2*‐280/292A mutant. Immunofluorescence was conducted on transfected HEK293 cells, and immunofluorescence images were acquired via laser confocal fluorescence microscopy (Figure 4a). As seen in the confocal images, POU6F2 was localized in the nucleus, which also implies that it is a transcriptional regulator, as previously determined. Moreover, the confocal images, indicate that the expression level of mutant *POU6F2* is lower than that of wild‐type POU6F2. In addition, we investigated POU6F2 protein expression by western blotting in the HEK293 and MMQ cell lines. We extracted protein from MMQ cells transfected with *POU6F2* wild‐type and mutant plasmids and subjected the lysates to western blotting (Figure 4b). The immunofluorescence and western blot results showed that the expression level of the mutant POU6F2 protein was significantly lower than that of wild‐type POU6F2. We then performed MTS assays to evaluate cell proliferation and found that overexpression of POU6F2 inhibited MMQ cell viability (Figure 4c), but this inhibitory effect was abolished by the *POU6F2* 280/292 mutation. To determine whether overexpression of POU6F2 affects the ability of MMQ cells to secrete PRL, we measured the level of PRL in the supernatant of MMQ cells using a PRL ELISA kit. The ELISA results suggested that overexpression of POU6F2 inhibited the ability of MMQ cells to secrete PRL (Figure 4d). Taken together, our results indicate that overexpression of POU6F2 inhibits cell viability and decreases PRL levels in prolactinomas and that the Pro280/Asn292 residues might play an important role in POU6F2 function.

## 4 DISCUSSION

PRL‐secreting adenomas are monoclonal in nature, supporting the theory that a spontaneous somatic mutation is the primary pathogenetic mechanism in this disorder (Herman, Fagin, Gonsky, Kovacs, & Melmed, 1990). However, genetic lesions have seldom been reported in these tumors. Our current study shows that the number of somatic mutations in prolactinomas is low. We found that a patient with prolactinoma had a higher tumor mutational burden than other prolactinoma patients. Many somatic mutations were found in the tumor tissues of this patient with prolactinoma, including Dmx like 2 (*DMXL2*), cytochrome P450 family 4 subfamily B member 1 (*CYP4B1*), Epidermal growth factor (*EGF*), mutY DNA glycosylase (*MUTYH*), radial spoke head 6 homolog A (*RSPH6A*), solute carrier organic anion transporter family member 1B3 (*SLCO1B3*), and *POU6F2*. *DMXL2* and *POU6F2* were confirmed by sanger sequencing. *DMXL2* could encode proteins which can regulate the Notch signaling pathway. Some researchers point out that the Arg2417His variant in DMXL2 is associated with dominant nonsyndromic hearing loss (Chen et al., 2017).

The somatic *POU6F2* mutation was selected for further research because it is a biallelic mutation and belongs to the POU family like POU domain, class 1, transcription factor 1 (*POU1F1*), also named pituitary transcript factor 1 (Pit‐1). Pit‐1 is a pituitary‐specific transcription factor involved in the generation, differentiation, and proliferation of three pituitary cell types: lactotrophs, somatotrophs, and thyrotrophs (Pellegrini et al., 1994).

*POU6F2* is a member of the POU family that is involved in cell type‐specific differentiation (Phillips & Luisi, 2000; Rosenfeld, 1991). However, very few reports on *POU6F2*. *POU6F2* was originally cloned from the human retina and is also known as retina‐derived POU‐domain factor‐1 (Zhou, Yoshioka, & Nathans, 1996). *POU6F2* plays a very important role in corneal development and is a potential risk factor for glaucoma in humans (King et al., 2018). POU6F2 has been reported to be expressed also in the developing midbrain (Zhou et al., 1996), pituitary (Yoshida et al., 2014), and kidneys (Di Renzo et al., 2006). POU6F2 expression was reported to be upregulated during corneal endothelial cell differentiation. However, some researchers found POU6F2 expression was decreased during neural and renal differentiation (Di Renzo et al., 2006; Yoshida et al., 2014). *POU6F2* is the second most frequently mutated gene in low‐grade mucoepidermoid carcinomas (Kang et al., 2017). Another study showed that this gene is a tumor suppressor and is involved in hereditary predisposition to Wilms' tumor (Miozzo et al., 1996; Perotti & Giovanna De Vecchi, 2004). To investigate the functions of *POU6F2*, some researchers generated an inducible stable transfect from HEK293 cells and showed that POU6F2 reduced cell proliferation and in vivo tumor growth (Fiorino et al., 2016), consistent with our results.

Although our study shows that POU6F2 can affect cell viability and PRL secretion ability in prolactinomas, we did not clarify the mechanism by which POU6F2 acts in prolactinomas. Thus, further research should be performed to explore the action mechanism of POU6F2 in prolactinomas. Some studies have shown that POU6F2 is expressed in the stem/progenitor cells of the rat pituitary primordium as well as in the diencephalon and retina. In addition, POU6F2 is abundantly expressed in early embryonic periods, followed by a decrease during pituitary development, indicating that this factor plays a role in pituitary cell differentiation (Yoshida et al., 2014). Perhaps there are some internal connections between POU6F2 and differentiation that have yet to be revealed. In this study, we used plasmids and siRNA to overexpress and knock down POU6F2 in the MMQ cell line and found that the viability and PRL secretion ability of MMQ cells with POU6F2 overexpression were decreased, but the viability and PRL secretion ability of MMQ cells with POU6F2 knockdown were increased. This pattern suggests that *POU6F2* is a tumor suppressor in prolactinomas.

In conclusion, our results provide the first indication that a novel biallelic *POU6F2* mutation may cause prolactinomas and that *POU6F2* is a tumor suppressor in prolactinomas. The mutation of *POU6F2* plays an important role in some prolactinomas, and *POU6F2* may be a promising target for developing new therapies against prolactinomas.

## CONFLICTS OF INTEREST

All authors declare no potential conflicts of interest.
