# Supplementary material for: Identification of a novel somatic mutation of POU6F2 by whole‐genome sequencing in prolactinoma
Source: Mol Genet Genomic Med. 2019 Nov 6;7(12):e1022. doi: 10.1002/mgg3.1022 (PMC6900357; doi:10.1002/mgg3.1022)
Supplement: Supplementary file 1 [file MGG3-7-e1022-s001.docx]

**Supplementary Material**

Table S1 Clinical characteristics of the prolactinoma patient in this study.

| Age | Sex | Tumor size | Knosp | Pro-operative  plasma prolactin  levels (ng/ml) | Post-operative  plasma prolactin  levels (ng/ml) | Dopamine agonist  sensitivity | Follow-up time  from surgery  (months)/outcome |
| --- | --- | --- | --- | --- | --- | --- | --- |
| 43 | M | giant | IV | 1068 | 273 | resistant | 50/ persistence |

Table S2 Sequence of siRNA

| **Gene** | **Sequence** |
| --- | --- |
| *Pou6f2-Rat-674-sense*  *Pou6f2-Rat-674-antisense* | 5'- CCGCGAAUCUCACAAACAUTT -3'  5'- AUGUUUGUGAGAUUCGCGGTT -3' |
| *Pou6f2-Rat-725-sense*  *Pou6f2-Rat-725-antisense* | 5'- GCAUUAUGACUCUGCCAUUTT -3'  5'- AAUGGCAGAGUCAUAAUGCTT -3' |
| *Pou6f2-Rat-1145-sense*  *Pou6f2-Rat-1145-antisense* | 5'- CCAUUCCACUGAUGCCUAATT -3'  5'- UUAGGCAUCAGUGGAAUGGTT -3' |
| Negative control-sense  Negative control-antisense | 5'-UUCUCCGAACGUGUCACGUTT-3'  5'-ACGUGACACGUUCGGAGAATT-3' |
